# Supplementary material for: Chromothripsis during telomere crisis is independent of NHEJ, and consistent with a replicative origin
Source: Genome Res. 2019 May;29(5):737–49. doi: 10.1101/gr.240705.118 (PMC6499312; doi:10.1101/gr.240705.118)
Supplement: Supplemental Material [file supp_gr.240705.118_Supplemental_file_1.zip › contigs/annotated_contigs/DB56/contig.2.DB56_length_275_mean_cov_4.52.docx]

**DB56_length_275_mean_cov_4.52**

```
CCCTAGAAGAAAATCTAGGCAATACCATTCAGGATTTAGGCATGGACAAAGATCTCATGGTGAAATTGCCAAAAGCAATTGCAACAAAA
 >chr20:44356941-44357070 + E=2e-63 p=0e+00
ATAAATAAATAGCAAAATTAA|AAAAGCAATTGCAACAAAA|ATAAATAAATAGCAAAATTAAAAAAGCAATTGCAACAAAAGCAAAAA
                      >chr20:44357011-44357153 + E=3e-65
TTGACAAATGAGATCCAATTAAACTAAAGAGCTTCTGCACAGCAAAAAAAAAACTATCATCAGACAGAACAGGCAA|ATTAAATAAAGG

GAGAAAAATTT
```
